# Tau ablation rescues vascular amyloid‐related deficits in a cerebral amyloid angiopathy model

**DOI:** 10.1002/alz.092412

**Published:** 2025-01-03

**Authors:** Muriel D Mardones, Nur Jury, Enrique Chimal Juarez, Henika Patel, Jonathan Martinez, Katie Vanderbosch, Abigail Perkins, Yamil Marambio, Ruben Vidal, Cristian A Lasagna Reeves

**Affiliations:** ^1^ Stark Neurosciences Research Institute, Indiana University School of Medicine, Indianapolis, IN USA; ^2^ Department of Anatomy, Cell Biology, and Physiology, Indiana University School of Medicine, Indianapolis, IN USA; ^3^ Department of Pathology and Laboratory Medicine, Indiana University School of Medicine, Indianapolis, IN USA; ^4^ Center for Computational Biology and Bioinformatics, Indiana University School of Medicine, Indianapolis, IN USA

## Abstract

**Background:**

Close to 80 to 90% of subjects with AD also present cerebral amyloid angiopathy (CAA) a disease in which amyloid accumulation damages the vasculature an impairs blood flow. Since current AD therapies are targeting the disease focusing on amyloid, we are interested on determine how to decrease the accumulation of amyloid in the vasculature observed in CAA and our aim is to determine the impact of tau reduction in CAA pathogenesis.

**Method:**

We crossed the Tg‐FDD mice CAA model with *Mapt^‐/‐^* mice to decrease tau levels and analyzed the disease pathogenesis in the different genotypes though behavioral tests, histological and morphometric assays and transcriptomic analysis using the nCounter neuroimmflamation panel from Nanostring.

**Result:**

We determined that tau ablation improved motor strength in the Tg‐FDD mice model, reduced amyloid deposition in the vasculature, decrease fibrinogen levels in the cortex, reduced astrocyte branching process associated to immunoreactivity. Nanostring analysis revealed that microglia function, oligodendrocyte and cytokine signaling are altered in the Tg‐FDD mice and that in the Tg‐FDD, Mapt*^‐/‐^* mice there is an increase in this mechanisms restoring the values to the ones observed in wild type mice.

**Conclusion:**

We are currently evaluating the pathways observed in the distinct inflammatory profile in microglia and oligodendrocytes. Our results suggest that tau ablation decreased CAA pathology in the Tg‐FDD mice model, which shows the potential therapeutic implications of targeting tau in CAA and related neurodegenerative diseases

